# Association between sedentary time and metabolic syndrome: A cross-sectional study among Chinese Garze Tibetans

**DOI:** 10.3389/fpubh.2022.1009764

**Published:** 2022-11-17

**Authors:** Lei Guo, Yixuan Liu, Tingting Xue, Liang Liang, Yongcuo Nima, Yang Yang, Qun Li, Qiushi Zhang

**Affiliations:** ^1^Guangdong Second Provincial General Hospital, Guangzhou, China; ^2^Garze Tibetan Autonomous Prefecture People's Hospital, Kangding, China

**Keywords:** sedentary time, metabolic syndrome, Tibetans, Chinese, cross-sectional study

## Abstract

**Background:**

Chinese Tibetans have long hours of sitting without much physical activity given their religious behavior, raising potential harmful health hazards. However, the relationship between sedentary time and metabolic syndrome (MetS) has not been investigated in Chinese Tibetans.

**Methods:**

From Jan 2021 to Jun 2022, residents in Garze Tibetan Autonomous Prefecture in Southwest China's Sichuan province were recruited using a multi-stage, stratified, random-cluster sampling strategy. MetS were ascertained using definition proposed by the International Diabetes Federation. Associations between sedentary time and the prevalence of MetS in the total sample and by age and sex were estimated using logistic regression models.

**Results:**

Among 971 Chinese Tibetan participants (mean age 41.1 years and 73.8% female), 319 (32.9%) were diagnosed as having MetS. We found positive associations of sedentary time over 11 h per day with the prevalence of MetS in crude (OR: 1.23; 95% CI: 1.12–1.36, *p* < 0.001), age and sex adjusted (OR: 1.18; 95% CI: 1.08–1.29, *p* < 0.001), and fully adjusted (OR: 1.17; 95% CI: 1.08–1.29, *p* < 0.001) models, compared to those who had <8 h of sedentary time per day. Sensitivity analyses suggest consistent positive association between sedentary time and each metric of MetS.

**Conclusions:**

Sedentary time longer than 11 h per day is significantly associated with increased risk of MetS, suggesting that polices to advocate health education may alleviate the health burden of MetS among Tibetans in China.

## Introduction

Metabolic syndrome (MetS) is a constellation of metabolic disorders including dyslipidemia, elevation of arterial blood pressure, and impaired fasting glycemia (1, 2). Numerous organizations including the World Health Organization and International Diabetes Federation have acknowledged MetS as a major risk factor for cardiovascular diseases and diabetes. Accompanying the temporal trend of obesity across the world, the fast grow in incidence and prevalence of MetS poses a significant health challenge that requires prompt and effective plans to cope with (3–5).

Similar to the global surge of MetS, the incidence and prevalence of MetS have also been rapidly increasing in the past 20 years in China, owing to miraculous economic growth, expeditious urbanization, adoption of Western pattern diet and sedentary lifestyle (3, 5–8). Tibetans are a special minority Chinese ethnicity group who live in high-altitude, sparsely populated, and less economically developed Western areas (9). Residents in Tibet mostly believe in Tibetan Buddhism and have a long ritual practice of meditation and chanting, resulting in long hours of sitting without much physical activity and potential negative health impacts (10–12). However, there is little evidence on the potential health effects of sedentary lifestyle on MetS in Tibetans Chinese.

Characterizing the relationship between sedentary time and MetS among Chinese Tibetans helps gain insights into a minority group and socially disadvantaged population in Western China. This could further help marching toward the Sustainable Development Goals (SDG) 3 of ensuring healthy livelihood and improve the wellbeing for all people including those disadvantaged groups (13–17). In this large cross-sectional study including 971 residents who live in Garze Tibetan Autonomous Prefecture, Sichuan province, we collected numerous demographic, behavior, and laboratory measure data on the participants. We aim to understand the relationship between sedentary lifestyle and the prevalence of MetS, as well as potential effect modifications.

## Methods

### Sampling strategies and participants

**Figure 1:**
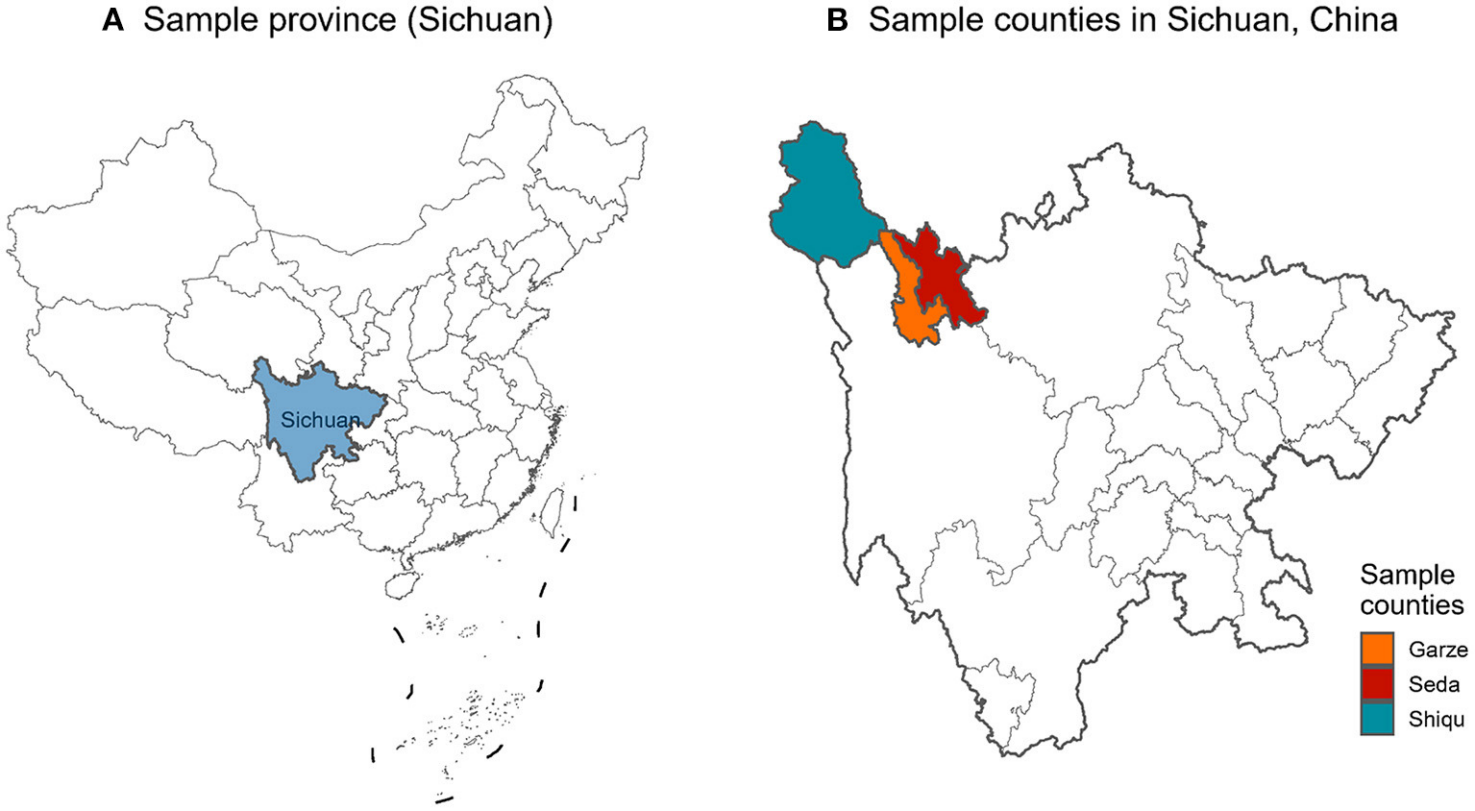
Geographic locations of the sample counties in Sichuan province, China. **(A)** Sample province (Sichuan). **(B)** Sample counties in Sichuan, China.

Garze Tibetan Autonomous Prefecture is in Southwest China's Sichuan province. From Jan 2021 to Jun 2022, a representative sample of individuals aged 18 years and over was enrolled to investigate the prevalence and risk factors of chronic disease. This investigation used a multi-stage, stratified, random-cluster sampling strategy. Three counties in the prefecture (Garze, Shiqu, and Seda) were selected first (Figure 1). In the second stage, Garze town in Garze county, Niga town in Shiqu county, and a Buddhist college (Larung Gar Buddhist Academy) in Seda county were randomly selected. All the eligible individuals 18 years and older were included. In total, we included 1,416 subjects with information about serum uric acid and medical history out of 3,093 subjects overall. This study was approved by the Ethics committee of Guangdong Second Provincial General Hospital (20201229-KJBF-02-01). All of the participants in this study signed an informed consent form.

### Data collection

A face-to-face interview was performed by trained doctors and nurses to collect demographic characteristics (age, sex, and education), lifestyle behaviors (smoking status, drinking status, and physical activity), anthropometric measurement (body mass index [BMI], waist circumference, and blood pressure). Education was divided into three levels (college or above, high school or equivalent, and less than high school). Physical activity was defined as ≥75 min of vigorous activity per week or ≥150 min of moderate activity per week (18). Smoking status and drinking status was classified into never or previous/current. Physical measurements (height, weight, and waist circumference) were measured by trained nurses. BMI was calculated by dividing the weight in kilograms by the height in meters squared. Blood pressure (including systolic blood pressure [SBP] and diastolic blood pressure [DBP]) of the participants was measured two times by a standardized automatic electronic sphygmomanometer after at least 5 min of rest in a seated position. A total of 5 mL venous blood samples of fasting blood were drawn by trained nurses in the morning and sent to the laboratory to evaluate the biochemical parameters, including comprising fasting blood glucose, triglycerides, total cholesterol (TC), LDL-cholesterol (LDL-C), and HDL-cholesterol (HDL-C).

### MetS

In line with the definitions made by the International Diabetes Federation and a few previous studies (19, 20), MetS is defined as people with abdominal obesity who meet at least two of the following four criteria: triglycerides ≥1.7 mmol/L; HDL-C < 1.03 mmol/L for male or <1.29 mmol/L in female; SBP ≥130 mmHg or DBP ≥85 mmHg; fasting glucose ≥5.6 mmol/L. Waist circumference was used to describe abdominal obesity (≥85 cm for male or ≥80 cm for female) according to Chinese guidelines (20).

### Sitting time

The sitting time was assessed according to the question “How many hours do you usually sit in 1 day?”. The response scale contained 4 options: (1) <8h; (2) 8–9 h; (3) 10–11 h; and (4) >11 h.

### Statistics analyses

Pearson Chi-square tests were used to explore the connection between categorical variables and the *T*-tests were applied to examine the differences of continuous variables between MetS groups in this study. We calculated the odds ratio (OR) for MetS by using a series of logistic regression models based on the sitting time and other covariables. Three models were conducted: crude model including sitting time only; basic model adjusted for age and sex; and fully adjusted model additionally accounted for the other independent variables, including education, smoking status, drinking status, BMI, and physical activity.

We further conducted subgroup analyses by sex (male and female) and age (<40 and ≥40) to examine potential effect modifications. The interaction term of the stratifying covariate with sitting time was implemented to test the differences between subgroups (21).

To test the robustness of our findings to each metric of MetS, we estimated the associations between sedentary time and each measure of MetS (abdominal obesity, triglycerides, HDL-C, hypertension, and hyperglycemia) in separate logistic regression models.

All statistical analyses were performed using R software, version 3.6.1. *P* < 0.05 was considered statistically significant.

## Results

### Sample characteristics and the prevalence of MetS

Table 1 shows the demographic information and laboratory test results of the 971 participants involved in this study and stratified by the status of Metabolic syndrome. Among the overall participants, 319 (32.9%) were diagnosed as having MetS according to our definitions. The participants were middle-aged (mean age 41.1 years), mostly female (73.8%), lack of education (72.9% did not receive high school education), high prevalence of previous or current smoking (32.9%), low prevalence of alcohol drinking (11%) and sedentary time <8 h (30.3%). The difference in demographic variables between participants who were included and excluded is shown in Supplementary Table S1. The participants who were included in the final analyses were slightly younger, had higher proportion of females and higher education.

**Table 1:** Characteristics of the overall study participants and by metabolic syndrome.

**Characteristics**	**Overall participants** **(*N* = 971)**	**Metabolic syndrome (MetS)**		
		**No (*N* = 652, 67.1%)**	**Yes (*N* = 319, 32.9%)**	***p*-value**
Sedentary time: *n* (%), hours per day				<0.001
<8	294 (30.3)	212 (32.5)	82 (25.7)	
8–9	402 (41.4)	277 (42.5)	125 (39.2)	
10–11	138 (14.2)	93 (14.3)	45 (14.1)	
>11	137 (14.1)	70 (10.7)	67 (21.0)	<0.001
Age: mean (SD), years	41.1 (13.5)	37.6 (12.0)	48.1 (13.8)	<0.001
Sex: *n* (%)				0.002
Male	254 (26.2)	150 (23.0)	104 (32.6)	
Female	717 (73.8)	502 (77.0)	215 (67.4)	
Education level: *n* (%)				<0.001
College or above	65 (6.7)	50 (7.7)	15 (4.7)	
High school or equivalent	198 (20.4)	132 (20.2)	66 (20.7)	
Less than high school	708 (72.9)	470 (72.1)	238 (74.6)	
Smoking: *n* (%)				<0.001
Never	564 (58.1)	424 (65.0)	140 (43.9)	
Previous or current	407 (41.9)	228 (35.0)	179 (56.1)	
Drinking: *n* (%)				0.002
Never	864 (89.0)	595 (91.3)	269 (84.3)	
Previous or current	107 (11.0)	57 (8.7)	50 (15.7)	
Physical activity: *n* (%)				<0.001
Yes	652 (67.1)	452 (69.4)	200 (62.7)	
No	319 (32.9)	200 (30.6)	119 (37.3)	
BMI: mean (SD), kg/m^2^	24.5 (5.4)	23.1 (4.9)	27.3 (5.2)	<0.001
Waist circumference: mean (SD), cm	84.3 (10.2)	81.3 (9.7)	94.1 (11.1)	<0.001
SBP: mean (SD), mmHg	120.7 (18.0)	113.8 (13.5)	134.9 (17.7)	<0.001
DBP: mean (SD), mmHg	78.1 (12.7)	73.6 (9.8)	87.3 (12.9)	<0.001
Fasting blood glucose: mean (SD), mmol/L	5.3 (1.5)	4.9 (1.3)	6.2 (1.7)	<0.001
Triglycerides: mean (SD), mmol/L	1.4 (0.9)	1.2 (0.6)	1.9(1.3)	0.010
Total cholesterol: mean (SD), mmol/L	5.0 (2.3)	4.8 (2.6)	5.4 (1.0)	<0.001
HDL- cholesterol: mean (SD), mmol/L	1.4 (0.4)	1.4 (0.4)	1.5 (0.4)	0.34
LDL- cholesterol: mean (SD), mmol/L	2.9 (0.9)	2.8 (1.0)	3.1 (1.1)	<0.001

BMI, body mass index; DBP, diastolic blood pressure; HDL, high-density lipoprotein; LDL, low-density lipoprotein; SBP, systolic blood pressure; SD, standard deviation.

Compared to participants who did not have MetS, those with MetS had a higher prevalence of sedentary time >11 h (21.0% in those with MetS and 10.7 in those without MetS); participants with MetS were older, had more male, had a lower level of education, a higher prevalence of smoking and alcohol drinking, and less physical activity. In addition, participants with MetS had much higher BMI and larger waist circumference, higher levels of diastolic and systolic blood pressure, fasting blood pressure, triglycerides, total cholesterol and low-density lipoprotein, compared to those without MetS.

### Association between sedentary time and MetS in Chinese Tibetans

Table 2 demonstrates the association between sedentary time and the prevalence of MetS in crude, age and sex adjusted, and fully adjusted models. Compared to participants who had <8 h of sedentary time, we observed significant positive associations of sedentary time over 11 h per day with the prevalence of MetS in crude (OR: 1.23; 95% CI: 1.12–1.36), age and sex adjusted (OR: 1.18; 95% CI: 1.08–1.29), and fully adjusted (OR: 1.17; 95% CI: 1.08–1.29) models. The p for nonlinear trend statistics were all significant, indicating the associations between sedentary time and MetS were not linear. This was supported by the much smaller OR estimates and nonsignificant *p*-values for sedentary time categories of 8–9 and 10–11 h per day.

**Table 2:** Association between sedentary time and metabolic syndrome in 971 Tibetans in Garze Tibetan Autonomous Prefecture, Sichuan province, China.

**Sedentary time** **(hours per day)**	**Crude model**		**Basic model^†^**		**Fully adjusted model^‡^**	
	**OR (95% CI)**	***P* value**	**OR (95% CI)**	***P* value**	**OR (95% CI)**	***P* value**
<8	1 (ref.)	-	1 (ref.)	-	1 (ref.)	-
8–9	1.03 (0.96, 1.11)	0.370	1.03 (0.97, 1.10)	0.325	1.04 (0.98, 1.11)	0.192
10–11	1.04 (0.95, 1.15)	0.327	1.03 (0.94, 1.13)	0.491	1.02 (0.93, 1.11)	0.669
>11	1.23 (1.12, 1.36)	<0.001	1.18 (1.08, 1.29)	<0.001	1.17 (1.08, 1.29)	<0.001
*P*-values for nonlinear trend		<0.001		<0.001		<0.001

OR, odds ratio.

† The basic model adjusted for age and sex.

‡ The fully adjusted model adjusted for age, sex education, smoking status, drinking status, and BMI.

### Associations between sedentary time and MetS by age and sex

The associations between sedentary time and the prevalence of MetS by age group and sex are shown in Table 3. We can still observe a consistent pattern of sedentary time > 11 h per day having the largest effect sizes in different sex and age groups. Sedentary time > 11 h per day were statistically significant among female (OR: 1.19; 95% CI: 1.08–1.31), age <40 (OR: 1.13; 95% CI: 1.01, 1.27), and age ≥40 (OR: 1.20; 95% CI: 1.06–1.37) groups, but insignificant among male group, likely due to the small sample size (*N* = 254) in this group. The magnitude of association between sedentary time > 11 h per day and MetS was slightly stronger in female than in male and in age ≥40 than in age <40, but the *p*-values for interaction tests were insignificant.

**Table 3:** Association between sedentary time and metabolic syndrome by sex and age group in 971 Tibetans in Garze Tibetan Autonomous Prefecture, Sichuan province, China.

**Sedentary time** **(hours/d)**	**OR (95% CI)**		***P* for interaction**	**OR (95% CI)**		***P* for interaction**
	**Male (*n* = 254)**	**Female (*n* = 717)**		**Age <40 (*n* = 476)**	**Age ≥40 (*n* = 495)**	
<8	1 (ref.)	1 (ref.)	0.55	1 (ref.)	1 (ref.)	0.71
8–10	1.01 (0.92, 1.12)	1.02 (0.85, 1.22)		1.04 (0.97, 1.14)	1.03 (0.94, 1.15)	
10–11	1.04 (0.97, 1.12)	1.05 (0.92, 1.20)		1.02 (0.91, 1.15)	1.01 (0.89, 1.15)	
>11	1.12 (0.93, 1.35)	1.19 (1.08, 1.31)		1.13 (1.01, 1.27)	1.20 (1.06, 1.37)	

### Sensitivity analyses

To test the robustness of the findings on the association between sedentary time and MetS at specific MetS measurements, we further constructed logistic regression models for the five metrics of MetS (Table 4). We found that sedentary time >11 h were significantly associated with increased risk of abdominal obesity (OR: 1.11, 95% CI: 1.01–1.21), triglycerides (OR: 1.14, 95% CI: 1.03–1.25), HDL-C (OR: 1.18, 95% CI: 1.08–1.29), and hyperglycemia (OR: 1.15, 95% CI: 1.01–1.29), while the association was not significant for hypertension (OR: 1.06, 95% CI: 0.97–1.16). Overall, the trend of positive association between sedentary time >11 h and MetS was consistent at different metrics of MetS.

**Table 4:** Association between sedentary time and components of metabolic syndrome in 971 Tibetans in Garze Tibetan Autonomous Prefecture, Sichuan province, China.

**Sedentary time** **(hours per day)**	**OR (95% CI)**				
	**Abdominal obesity^a^**	**Triglycerides^b^**	**HDL-C^c^**	**Hypertension^d^**	**Hyperglycemia^e^**
<8	1 (ref.)	1 (ref.)	1 (ref.)	1 (ref.)	1 (ref.)
8–9	1.01 (0.94, 1.08)	1.01 (0.96, 1.07)	1.03 (0.97, 1.10)	0.97 (0.89, 1.06)	0.98 (0.96, 1.01)
10–11	1.03 (0.94, 1.13)	1.01 (0.94, 1.09)	1.03 (0.94, 1.13)	1.03 (0.96, 1.10)	1.02 (0.93, 1.11)
>11	1.11 (1.01, 1.21)	1.14 (1.03, 1.25)	1.18 (1.08, 1.29)	1.06 (0.97, 1.16)	1.15 (1.01, 1.29)

a Waist circumference ≥85 cm for male or ≥80 cm for female.

b Triglycerides ≥ 1.7 mmol/L.

c HDL-C < 1.03 mmol/L for male or < 1.29 mmol/L in female.

d SBP ≥130 mmHg or DBP ≥ 85 mmHg.

e Fasting glucose ≥ 5.6 mmol/L.

## Discussion

In this large cross-sectional study of 971 participants in Garze Tibetan Autonomous Prefecture of Sichuan, China, 319 (32.9%) were diagnosed as having MetS, and we found that sedentary time > 11 h per day were associated with 17% (95% CI: 8–29%) increase in the odds of MetS, compared to those with sedentary time <8 h per day. The findings were consistent across age and sex groups. To our knowledge, this is the first study that investigates the relationship between sedentary time and MetS among Chinese Tibetans, who reside in rural western China at high altitudes, live in poverty, and hold longer ritual sedentary time influenced by their religious practice.

With the rapid economic development in the last 30 years, China has witnessed substantial social, behavior, lifestyle, and environmental transitions, which lead to a drastic surge in the prevalence of obesity and associated metabolic disorders (22). Although China's national “dynamic zero COVID-19” policy since 2020 has effectively and dramatically reduced the transmission and mortality of COVID-19 (23–25), obesity, mental health, and stagnation of economic growth caused by lockdown, segregation, and mass nucleic acid testing are projected to have substantial synergistic effects on the prevalence of MetS and associated health consequences in Chinese citizens (26–28), including Chinese Tibetans. Although this is a cross-sectional study that could not infer direct causal effects of sedentary time on MetS, but it appears that long sedentary time over 11 h per day is a significant predictor of MetS among Chinese Tibetans, which suggests that reducing sedentary time may help alleviate the burden of MetS among this vulnerable group.

There are a few investigations on the association between sedentary time and metabolic syndrome in China and in other countries. For example, the Nantong Metabolic Syndrome Study reported that 21.6% participant had MetS, and vigorous physical activity was associated with 15–40% decreased odds of metabolic syndrome (29). Another nationwide study of 4,865 adults from the China Health and Nutrition Surveys reported that sedentary time was associated with significantly higher risk of MetS (OR: 1.3; 95% CI: 1.1–1.6) (30). An earlier meta-analysis reviewed 10 cross-sectional studies (*n* = 21,393) reported that more sedentary time was associated with an increased odds of MetS (OR 1.73, 95% CI 1.55–1.94) (31). Compared to these previous investigations, our study reported consistently positive association but with a lower OR (OR: 1.17; 95% CI: 1.08–1.29). More importantly, our study filled in a knowledge gap of the relationship between sedentary behavior and MetS among Tibetans in China, and the results have policy implications on health equity.

Although our data were collected from a Tibetan-dominant prefecture in Southwest China, several findings are consistent with those in other studies conducted in different areas, which corroborates the validity of our findings. The MetS prevalence of 32.9% in this study is similar to the results of a study among a group older Tibetans in Jiarong (Northwest Sichuan Province) using similar diagnosis criteria, which reported the MetS prevalence of 37.6% (20). Other studies using the China Multi-Ethnic Cohort reported the prevalence of MetS of 17.8% among Tibetans while 11.9% among both Tibetans and Han Chinese using a stricter National Cholesterol Education Program Adult Treatment Panel III (ATP III) criteria (32, 33). The difference in prevalence rate of MetS among Tibetans and a mixture of Han Chinese also reveals the higher prevalence of MetS in minority group Tibetans than the dominate group Han Chinese, suggesting further policy actions to improve health equity in Chinese minority ethnicity groups residing in remote rural areas (34).

Our study has several limitations. First, our study site includes only one prefecture in Sichuan, China, which limits the generalizability of findings to Tibetans in other parts of China. Second, our regression models included a limited set of covariates due to limited data accessibility and quality, which may lead to potential missing covariate bias in our findings. Third, this is a cross-sectional survey that lacks imposed treatment or intervention, which refrains from making causal claims about the relationship between sedentary time and MetS. Fourth, there are several definitions for MetS including those made by the World Health Organization in 1999, National Cholesterol Education Program ATP3 in 2005, and the one by the International Diabetes Federation in 2006 (used in our study); there is not yet consensus on the best definition of MetS, and the uncertainty in MetS definition may cause outcome misclassification and heterogeneity of results across studies. Fifth, sedentary time was collected as category variable and the cutoffs were somewhat arbitrary, and we were not able to construct exposure-response curves in the results.

Nonetheless, this is the first study that investigate the relationship between sedentary time and MetS among Chinese Tibetans. Our study recruited a relatively large sample of about 1,000 middle-aged Tibetan participants using a multi-stage, stratified, random-cluster sampling strategy, and collected a set of variables involving demographics, behavior, and laboratory tests. The findings fill the knowledge gap on the magnitude of association between sedentary time and MetS among Chinese Tibetans, suggesting local efforts to educate Tibetans to boost physical activity and reduce sedentary time to reduce the prevalence of MetS, as well as the resulting cardiovascular health outcomes.

## Conclusions

In this large cross-sectional survey of Chinese Tibetans in Sichuan Province, around one third of the participants manifest evidence of MetS, showing high burden of MetS among Chinese Tibetans. Sedentary time longer than 11 h per day is significantly associated with increased risk of MetS. The findings suggest that efforts on health education may alleviate the health burden of MetS among Tibetans in China.

## Data availability statement

The datasets presented in this article are not readily available because please contact author for data requests. Requests to access the datasets should be directed to QZ, zhangqsh@gd2h.org.cn.

## Ethics statement

This study was approved by the Ethics Committee of Guangdong Second Provincial General Hospital (20201229-KJBF-02-01). All of the participants in this study signed an informed consent form.

## Author contributions

LG: conceptualization, investigation, visualization, writing—original draft, and writing—reviewing and editing. YL: investigation, visualization, writing—original draft, and writing—reviewing and editing. TX, LL, YN, YY, and QL: investigation. QZ: investigation, visualization, and supervision. All authors contributed to the article and approved the submitted version.

## Funding

This study was supported by the Guangzhou Municipal Science and Technology Project (202108050520130003 and 202108050520130004) and 3D Printing Research Grant of Guangdong Second Provincial General Hospital (3D-B2020013).

## Conflict of interest

The authors declare that the research was conducted in the absence of any commercial or financial relationships that could be construed as a potential conflict of interest.

## Publisher's note

All claims expressed in this article are solely those of the authors and do not necessarily represent those of their affiliated organizations, or those of the publisher, the editors and the reviewers. Any product that may be evaluated in this article, or claim that may be made by its manufacturer, is not guaranteed or endorsed by the publisher.

## References

[1] Eckel RH Grundy SM Zimmet PZ. The metabolic syndrome. Lancet. (2005) 365:1415–28. 10.1016/S0140-6736(05)66378-7 15836891

[2] Kassi E Pervanidou P Kaltsas G Chrousos G. Metabolic syndrome: definitions and controversies. BMC Med. (2011) 9:48. 10.1186/1741-7015-9-48 21542944 PMC3115896

[3] Despres JP Lemieux I. Abdominal obesity and metabolic syndrome. Nature. (2006) 444:881–7. 10.1038/nature05488 17167477

[4] Grundy SM. Metabolic syndrome pandemic. Arterioscler Thromb Vasc Biol. (2008) 28:629–36. 10.1161/ATVBAHA.107.151092 18174459

[5] Saklayen MG. The global epidemic of the metabolic syndrome. Curr Hypertens Rep. (2018) 20:12. 10.1007/s11906-018-0812-z 29480368 PMC5866840

[6] Liu M Wang J Jiang B Sun D Wu L Yang S. Increasing prevalence of metabolic syndrome in a Chinese elderly population: 2001-2010. PLoS ONE. (2013) 8:e66233. 10.1371/journal.pone.0066233 23824753 PMC3688874

[7] Huang J Huang J Withers M Chien K Trihandini I Elcarte E. Prevalence of metabolic syndrome in Chinese women and men: a systematic review and meta-analysis of data from 734 511 individuals. Lancet. (2018) 392:S14. 10.1016/S0140-6736(18)32643-6

[8] Wang Y Mi J Shan XY Wang QJ Ge KY. Is China facing an obesity epidemic and the consequences? The trends in obesity and chronic disease in China. Int J Obes (Lond). (2007) 31:177–88. 10.1038/sj.ijo.0803354 16652128

[9] Cai M Lin X Wang X Zhang S Qian ZM McMillin SE. Ambient particulate matter pollution of different sizes associated with recurrent stroke hospitalization in China: a cohort study of 1.07 million stroke patients. Sci Total Environ. (2022) 856(Pt 2):159104. 10.1016/j.scitotenv.2022.159104 36208745

[10] Xue T Chiao B Xu T Li H Shi K Cheng Y. The heart-brain axis: a proteomics study of meditation on the cardiovascular system of Tibetan Monks. EBioMedicine. (2022) 80:104026. 10.1016/j.ebiom.2022.104026 35576643 PMC9118669

[11] Liu K Xu Y Wang S Shi R Gong S Li X. Buddhist Activities related to Sedentary behavior and Hypertension in Tibetan monks. J Hum Hypertens. (2019) 33:756–62. 10.1038/s41371-018-0136-0 30420645

[12] Xu J Yang Y Li Z Tashi N Sharma R Fang J. Understanding land use, livelihoods, and health transitions among Tibetan nomads: a case from Gangga Township, Dingri County, Tibetan Autonomous Region of China. Ecohealth. (2008) 5:104–14. 10.1007/s10393-008-0173-1 18787914

[13] Anderson I Bang A Bjertness E Connolly M King A Robson B. Indigenous and tribal peoples' health (The Lancet-Lowitja Institute Global Collaboration): a population study. Lancet. (2016) 388:131–57. 10.1016/S0140-6736(16)32465-5 27108232

[14] Tan X Wu Q Shao H. Global commitments and China's endeavors to promote health and achieve sustainable development goals. J Health Popul Nutr. (2018) 37:8. 10.1186/s41043-018-0139-z 29650054 PMC5898031

[15] Chen S Guo L Wang Z Mao W Ge Y Ying X. Current situation and progress toward the 2030 health-related sustainable development goals in China: a systematic analysis. PLoS Med. (2019) 16:e1002975. 10.1371/journal.pmed.1002975 31743352 PMC7340487

[16] Naidoo R Fisher B. Reset sustainable development goals for a pandemic world. Nature. (2020) 583:198–201. 10.1038/d41586-020-01999-x 32632244

[17] Cai M Liu E Bai P Zhang N Wang S Li W. The chasm in percutaneous coronary intervention and in-hospital mortality rates among acute myocardial infarction patients in rural and urban hospitals in China: a mediation analysis. Int J Public Health. (2022) 67:1604846. 10.3389/ijph.2022.1604846 35872707 PMC9302370

[18] Wang X Qian ZM Zhang Z Cai M Chen L Wu Y. Population attributable fraction of lung cancer due to genetic variants, modifiable risk factors, and their interactions: a nationwide prospective cohort study. Chemosphere. (2022) 301:134773. 10.1016/j.chemosphere.2022.134773 35500626

[19] Holt RI. International Diabetes Federation re-defines the metabolic syndrome. Diabetes Obes Metab. (2005) 7:618–20. 10.1111/j.1463-1326.2005.00519.x 16050956

[20] Li T Tang X Liu Y Li Y He B. Dietary patterns and metabolic syndrome among urbanized Tibetans: a cross-sectional study. Environ Res. (2021) 200:111354. 10.1016/j.envres.2021.111354 34102164

[21] Wang R Ware JH. Detecting moderator effects using subgroup analyses. Prev Sci. (2013) 14:111–20. 10.1007/s11121-011-0221-x 21562742 PMC3193873

[22] Pan XF Wang L Pan A. Epidemiology and determinants of obesity in China. Lancet Diabetes Endocrinol. (2021) 9:373–92. 10.1016/S2213-8587(21)00045-0 34022156

[23] Cai J Deng X Yang J Sun K Liu H Chen Z. Modeling transmission of SARS-CoV-2 Omicron in China. Nat Med. (2022) 28:1468–75. 10.1038/s41591-022-01855-7 35537471 PMC9307473

[24] Cai J Hu S Lin Q Ren T Chen L. China's ‘dynamic zero COVID-19 strategy' will face greater challenges in the future. J Infect. (2022) 85:e13–4. 10.1016/j.jinf.2022.04.025 35447231 PMC9015719

[25] Pan A Liu L Wang C Guo H Hao X Wang Q. Association of Public Health Interventions With the Epidemiology of the COVID-19 Outbreak in Wuhan, China. JAMA. (2020) 323:1915–23. 10.1001/jama.2020.6130 32275295 PMC7149375

[26] Jia P Zhang L Yu W Yu B Liu M Zhang D. Impact of COVID-19 lockdown on activity patterns and weight status among youths in China: the COVID-19 Impact on Lifestyle Change Survey (COINLICS). Int J Obes (Lond). (2021) 45:695–9. 10.1038/s41366-020-00710-4 33277588 PMC7715639

[27] Zhou J Liu L Xue P Yang X Tang X. Mental health response to the COVID-19 outbreak in China. Am J Psychiatry. (2020) 177:574–5. 10.1176/appi.ajp.2020.20030304 32375540

[28] Zhang N Hu X Zhang Q Bai P Cai M Zeng TS. Non-high-density lipoprotein cholesterol: High-density lipoprotein cholesterol ratio is an independent risk factor for diabetes mellitus: results from a population-based cohort study. J Diabetes. (2018) 10:708–14. 10.1111/1753-0407.12650 29437292

[29] Xiao J Shen C Chu MJ Gao YX Xu GF Huang JP. Physical activity and sedentary behavior associated with components of metabolic syndrome among people in rural China. PLoS ONE. (2016) 11:e0147062. 10.1371/journal.pone.0147062 26789723 PMC4720370

[30] Bai J Wang Y Zhang X Ouyang Y Zhang B Wang Z. Associations of sedentary time and physical activity with metabolic syndrome among Chinese adults: results from the China health nutrition survey. Biomed Environ Sci. (2021) 34:963–75. 10.3967/bes2021.132 34981719 PMC10023150

[31] Edwardson CL Gorely T Davies MJ Gray LJ Khunti K Wilmot EG. Association of sedentary behaviour with metabolic syndrome: a meta-analysis. PLoS ONE. (2012) 7:e34916. 10.1371/journal.pone.0034916 22514690 PMC3325927

[32] Li K Zhang Q Cai H He R Nima Q Li Y. Association of Tibetan Habitual food and metabolic syndrome among Tibetan people in China: a cross-sectional study. Front Nutr. (2022) 9:888317. 10.3389/fnut.2022.888317 35811962 PMC9263562

[33] Xiao X Qin Z Lv X Dai Y Ciren Z Yangla Y. Dietary patterns and cardiometabolic risks in diverse less-developed ethnic minority regions: results from the China Multi-Ethnic Cohort (CMEC) Study. Lancet Reg Health West Pac. (2021) 15:100252. 10.1016/j.lanwpc.2021.100252 34528018 PMC8383007

[34] Cai M Liu E Li W. Rural vs. urban patients: benchmarking the outcomes of patients with acute myocardial infarction in Shanxi, China from 2013 to 2017. Int J Environ Res Public Health. (2018) 15:1930. 10.3390/ijerph15091930 30189602 PMC6165441

