# Supplementary material for: Association between sedentary time and metabolic syndrome: A cross-sectional study among Chinese Garze Tibetans
Source: Front Public Health. 2022 Nov 17;10:1009764. doi: 10.3389/fpubh.2022.1009764 (PMC9713937; doi:10.3389/fpubh.2022.1009764)
Supplement: Supplementary file 1 [file Table_1.DOCX]

Table S1. Distribution of population characteristics in individuals included and excluded in our analyses.

|  | Participants included in analyses  (N=971) | Participants excluded in analyses  (N=1,440) |
| --- | --- | --- |
| Age (years), mean (SD) | 41.1 (13.5) | 40.7 (14.2) |
| Sex (%) |  |  |
| Male | 254 (26.2) | 402 (27.9) |
| Female | 717 (73.8) | 1,038 (42.1) |
| Education (%) |  |  |
| College or above | 65 (6.7) | 86 (6.2) |
| High school or equivalent | 198 (20.4) | 270 (19.5) |
| Less than high school | 708 (72.9) | 1,031 (74.3) |

SD, standardized deviation
